# Scotch Physicians and Surgeons

**Published:** 1888-02-25

**Authors:** C. G. Furley


### Scotch Physicians and Surgeons.

By C. G. Furley.

### SIR ROBERT CHRISTISON.

Of all the professors that Edinburgh University has known, none was ever more distinctly and peculiarly a "member of the Senatus Academicus" than Sir Robert Christison. To him the university was most assuredly "the hub of all creation," as the American puts it; his pride and glory lay in its efficiency and prosperity; its enemies, or those whom he regarded as such, were his lifelong foes; and its interests bulked more largely in his estimation than any personal ones of his own. It was the creed in which he was born. The son of an Edinburgh professor, university deeds and misdeeds, university successes and failures, were the familiar interests of his youth; and throughout his eighty years of life they never lost their importance in his eyes. In an autobiography which he began on completing the fiftieth year of his professorship, he has, though he did not live to carry the record on beyond middle life, shown us clearly what manner of man he was; has pictured the proud, reserved, somewhat austere, but strictly honourable gentleman, with a sarcastic tongue and a scrutinising eye, combined with a loyal heart and a tender conscience, whom Edinburgh remembers with an affectionate respect, not unmixed with awe.

Robert Christison was born on the 18th of July, 1797. The mere fact of a man being born in the last century invests him with a certain peculiarity in our eyes. We have an unconfessed feeling that the boundary between the centuries is a high wall which it is in some way creditable to have overleaped. He who has heard the midnight chimes ring out 1799 and ring in 1800—we despise the paltry mathematical accuracy which begins a century with the year One instead of the year Nought—belongs, however meekly he may walk among us as if in no way distinguished from the rest of our species, to the romantic, the vaguely picturesque—to history as opposed to the gossip of to-day; history, for all its dignified airs, being in fact only the gossip of the day before yesterday. Thus, though Christison deprecates the possession of an exceptional memory in his vivid recollection of running up to his father's bed-room, when he was eight years old, to tell him of the death of Nelson and the victory of Trafalgar, we feel that the very lightness of tone in which he speaks of remembering those events removes him far from us. Nelson—Trafalgar: they are a part of the very long ago!

Robert Christison was the elder of twin sons. He seems to have thought that his twin brother John was intellectually his equal, and that it was only a natural modesty, developed by accident and education into diffidence, that made him contentedly pass his life as minister of a small country parish. For this excess of a virtue he blames their being brought up together. "It may be a pleasing idea, but it is a dubious measure," he protests, "to have twins constantly together at school and at play. The one who gets a little ahead of the other, as one of them is sure to do, is by parent and teacher unconsciously brought to the front." It was Robert who was thus brought to the front, if not by superior intellect, by superior energy of temperament. At school and at college he showed a quiet determination to take a creditable place—not the feverish passion of the bookworm, not the ambition of the self-seeker, but the self-respect of a man who owes it to his conscience and his family not to sit among the dunces.

Christison was full of family pride, though he alludes to his ancestry in somewhat sarcastic fashion. The Christisons came from the east coast of Scotland, and, as the name shows, were descended from some Norse Viking of invading fame; Sir Robert's mother was a Johnstone, and as her family, he says, "sealed with the Flying Spur, they must have claimed descent from the Border rievers of Annandale. With a pirate at one end of the line and a robber at the other, one may fairly pretend to a decent ancestry: 'There be land rats and there be water rats,' and I am come of both, probably."

His education proceeded on the orthodox lines of the schooling of Edinburgh youth in those days. He went to the High School, and was well grounded in the Latin tongue, besides receiving such an education in English as would seem small to a Board-school boy of our times, and a knowledge in all its severity of the rules of arithmetic. He never regretted the narrowness of his school curriculum; on the contrary, he was given to favourably comparing its results with those of more liberal systems. To teach a boy practical things—things that will help him in after-life—is a very good thing; but he doubts not only if Anglo-Saxon literature and physical geography tend more to that consummation than does the Æneid, but if boys between the ages of eight and fourteen will profit by many of the subjects now taught them. It is true, though we are only now beginning to learn it, that we cannot assuredly predict that a boy will grow up cultured and useful because he studied Euclid in the nursery, and that our schoolboys, with their smattering of every science and art and their real knowledge of no single one, as often develop into conceited and opinionated nonentities as into thoughtful men; but on this question of education it may be doubted if the time-honoured system of classical education is the best possible training of the observing and reasoning faculties. When Latin was the common tongue of all learned Europe there was a good and obvious reason for studying it which no longer exists. French is quite as useful at the present day; German opens up to us a literature rich in ideas of more guiding and suggestive value than all the tale of Horace's loves and Dido's sorrows; while the mediæval literatures of France and Italy offer to the more advanced student as much of interesting and curious as any classic tongue. The Latin class which Robert Christison's father taught in Edinburgh University is still called that of "humanity," but it has ceased to deserve the name; its claims to human sympathy have faded with the help it gave to human intercourse.

From school young Christison went to collegè, going through the arts classes with some distinction, and showing a special aptitude for mathematics. "At eighteen," he says, "I could fight my way through Newton's 'Principia,' and when, six years afterwards, my destiny seemed to be chemistry, and I therefore determined to master, if I could, the mathematical physics of the day, I found I could travel faithfully through the first volume of Biot's 'Traité de Physique.'"

The predilection for chemistry to which he thus alludes was the first direct step towards the fulfilment of his life work; but in 1813, when he began to study it, he had no desire to carry on a medical career in any of its branches. When his father asked him what occupation he would like to take up, he answered, "a civil engineer's." Professor Christison, the elder, looked askance at the suggestion. The profession of a civil engineer was not then a profitable one, nor was it one that commended itself to his ideas of dignity; and he persuaded Robert to give up the notion. The latter never regretted having forsaken his first choice of a calling to pursue that of medicine; though, recalling more than fifty years afterwards his father's anxiety lest he should not make his living as an engineer, he remarks that in view of the number of public works—lighthouses, tunnels, bridges—begun within ten years after he gave up the idea, he would not have been qualified long before the time when the demand for civil engineers far exceeded the supply.

He seems to have turned readily enough from his boyish fancy, and to have pursued his medical studies with all his characteristic energy, though when studying anatomy under Monro *tertius* it took him some time to get over his horror at the sight of dead bodies, and the feeling of sickness induced by the odour of the anatomy theatre. "This," he says, "may have been owing to what has proved a useful endowment in other circumstances—an acute and discriminating sense of smell. But at any rate the same possession has been a sad annoyance to me in the pursuit of anatomy and pathology—such even as to have often required a strong sense of duty to overcome it."

There are smells and smells, however, and there were some which do not seem to have appeared to Christison so objectionable. The odour of, say, sulphuretted hydrogen is not pleasing to most nostrils; but our student's sense of smell did not prevent his forming a small laboratory of his own for the purpose of verifying the experiments he witnessed in the chemistry class. Professor Hope, his teacher, was himself famed for the brilliancy and accuracy of his experiments, but he provided no practical class where his pupils could learn to emulate his feats. In default of this, Christison, with eleven fellow-students, one of whom was Syme, afterwards the well-known surgeon, formed themselves into a society for the purpose of repeating and practising the experiments they saw in class, in so far as their limited means and apparatus would permit. They made no discoveries and sought to make none, but their experiments taught them aptitude in handling apparatus, resource in difficulties, and promptitude in danger. There were, as might be expected when a dozen very young men meddle with chemicals, plenty of opportunity for exercising the last quality. On one occasion, when a suspicious effervescence in a closed flask gave fore-warning of an explosion, Chistison blew out the lights, called to the society to leave the room at once, and hid himself under the table to await results. His fellow-students had scarcely made themselves safe on the other side of the door when there came a burst, a roar, and a strong smell of malodorous gas; the apparatus distributed itself impartially over the room, which fortunately had no superfluity of furniture; but when, after some time, the society ventured timidly to open the door and investigate with a candle, Christison was found under the protecting table, safe and ready to renew experiments.

*(To be continued.)*

